# Proteomic characterization of *Lysinibacillus* reveals early-stage PET biodegradation potential

**DOI:** 10.3389/fmicb.2026.1802173

**Published:** 2026-03-24

**Authors:** Radoslaw B. Dudziak, Víctor Muñoz-Hisado, Andrea Hidalgo-Arias, María Martínez-Carrancho, Eva Garcia-Lopez, Farayde Matta Fakhouri, Emma Martinez-Alonso, Alberto Alcázar, Margrét Auður Sigurbjörnsdóttir, Gustavo Graciano Fonseca, Cristina Cid

**Affiliations:** 1 Centro de Astrobiología (CAB), CSIC-INTA, Madrid, Spain; 2 Faculty of Natural Resource Sciences, School of Business and Science, University of Akureyri, Akureyri, Iceland; 3 Escuela de Doctorado de la Universidad Autónoma de Madrid. Centro de Estudios de Posgrado, Ciudad Universitaria de Cantoblanco, Madrid, Spain; 4 Department of Materials Science and Engineering, Universitat Politècnica de Catalunya – UPC BarcelonaTech, Terrassa, Spain; 5 Departamento de Investigacion, Hospital Ramón y Cajal, Instituto Ramón y Cajal de Investigación Sanitaria, Madrid, Spain

**Keywords:** enzymatic hydrolysis, *Lysinibacillus*, microbial consortium, PET-degradation, plastic biodegradation, plastic pollution, proteomic profiling, sustainable waste management

## Abstract

Plastic pollution is a global challenge due to the persistence of synthetic polymers such as polyethylene terephthalate (PET) and the limited efficiency of current recycling strategies. While microbial biodegradation is a promising alternative, the relatively recent introduction of plastics has constrained microbial evolutionary adaptation and enzymatic efficiency. In this study, a PET-associated bacterial strain was isolated from Icelandic soil, identified as *Lysinibacillus* sp. via 16S rRNA sequencing, and evaluated through growth assays, proteomics, *in silico* screening, and surface imaging. In mineral medium with PET as the sole carbon source, *Lysinibacillus* sp. exhibited a shorter lag phase and higher early-stage growth than the reference strain *Ideonella* sakaiensis (*p* < 0.05 at Weeks 1, 2, 4, and 6) over six weeks. FE-SEM revealed microbial colonization, surface erosion, fissures, and delamination, indicating polymer surface alteration. MALDI-TOF MS proteomic analysis did not detect canonical PET-degrading enzymes such as PETase or MHETase. The *in silico* genome-wide screen similarly failed to identify PETases or other known polyester hydrolases carrying the conserved GXSXG motif, the Ser-His-Asp catalytic triad, or compatible α/β-hydrolase domain architecture. Instead, PET exposure triggered metabolic reprogramming dominated by oxidative-stress response proteins (peroxiredoxins, superoxide dismutases, thiol peroxidases) and central metabolic enzymes. Although several proteins annotated as hydrolases were expressed, these lacked the catalytic signatures and structural features characteristic of validated PET-degrading polyesterases. Taken together, the proteomic and *in silico* results indicate that *Lysinibacillus* sp. responds to PET through broad metabolic and oxidative-stress adaptation rather than through expression of dedicated PET-hydrolyzing enzymes. This stress-driven remodeling may support limited transformation of PET-associated compounds but does not constitute evidence of direct PET depolymerization. The rapid adaptive response of *Lysinibacillus* sp. complements the slower, enzyme-driven strategy of *I. sakaiensis*, supporting the potential of microbial consortia for multi-stage plastic biodegradation.

## 1 Introduction

Plastic pollution has emerged as a serious global environmental issue, driven largely by the excessive production of plastic materials and the limited capacity of current recycling systems. As a result, large quantities of plastic end up in landfills, oceans, and other ecosystems, where they persist for decades or even centuries. Meanwhile, plastic waste continues to fragment into smaller particles, forming microplastics and nanoplastics that have now infiltrated both terrestrial and aquatic ecosystems. These tiny particles pose a significant threat to biodiversity and have been found to enter food chains, raising concerns about their impact on animal and human health (Boctor et al., 2025).

In response to this growing crisis, scientists are actively investigating long-term solutions, one of which involves the use of microorganisms capable of breaking down plastic materials. However, since the widespread use of plastics only began in the 1950s, microbes have had relatively little time to evolve mechanisms to degrade these synthetic compounds effectively. Despite this, recent discoveries have offered hope. For instance, *I. sakaiensis*, has demonstrated the ability to degrade polyethylene terephthalate (PET), one of the most commonly used plastics (Yoshida et al., 2016). Similar bacteria with plastic-degrading capabilities have also been discovered in other parts of the world, including Pakistan and Germany, suggesting that microbial degradation could become a viable strategy if further developed and applied (Afreen et al., 2020).

One of the main challenges in addressing plastic pollution lies in the chemical stability of materials like PET and PE (polyethylene) (Supplementary Table 1). These plastics are characterized by their high molecular weight and hydrophobic nature, which make them particularly resistant to degradation through natural biological processes (Shah et al., 2008; Ishii et al., 2024). This resistance underscores the urgency of developing innovative solutions, such as microbial degradation, to mitigate the long-term environmental consequences of plastic pollution.

Numerous bacterial and fungal enzymes have been identified with the capacity to degrade synthetic polymers. Among the most notable are cutinases from *Thermobifida fusca* (Roth et al., 2014) and *Fusarium solani* (Müller et al., 2005). In particular, *F. solani* has demonstrated the ability to hydrolyze PET into its monomers through the action of its cutinase (Hellesnes et al., 2022). The cutinase from *Humicola insolens*, has also demonstrated the ability to degrade low-crystallinity PET, achieving up to 97% recovery of pure terephthalic acid (Quartinello et al., 2017).

Across multiple studies, elevated enzyme operating temperatures have emerged as a critical factor in enhancing PET degradation, as they increase the mobility of the amorphous fraction and thus its susceptibility to hydrolysis. However, prolonged exposure to high temperatures can induce microstructural changes in PET that hinder enzymatic degradation. To address this, the addition of catalysts to the reaction has been proposed as a strategy to facilitate degradation before the formation of resistant microstructures (Akram et al., 2024).

Under suitable environmental conditions, certain microorganisms are capable of producing enzymes that initiate the breakdown of synthetic polymers. Plastics such as PE and PET, widely used in packaging and textiles, are particularly resistant to degradation due to their high molecular weight and hydrophobic properties. However, recent advances in microbial biotechnology have demonstrated that enzymes such as laccases, peroxidases, and PET hydrolases (PETases) can facilitate the decomposition of these polymers into simpler, potentially reusable compounds (Tian et al., 2023). PET hydrolases are a class of enzymes that hydrolyze ester bonds within plastic polymers, converting them into simpler monomers. This process enables microorganisms to utilize these monomers as primary carbon sources, ultimately leading to further biodegradation and the release of end products such as CO_2_, H_2_O, CH_4_, and N_2_ (Khairul Anuar et al., 2022).

The growing concern over the environmental impact of large-scale plastic production has intensified research into the mechanisms and key enzymatic components involved in microbial PET degradation. Advances in genomics and protein engineering are expected to enable the development of PET hydrolases tailored for various applications. The biochemical degradation of plastics by bacteria is a multi-step process involving enzymatic hydrolysis, oxidation, and assimilation. Each stage is catalyzed by specific enzymes encoded in the bacterial genome. PET degradation typically begins with PETase, which hydrolyzes ester bonds to yield mono(2-hydroxyethyl) terephthalate (MHET), along with smaller amounts of terephthalic acid (TPA) and ethylene glycol (EG). MHETase then further hydrolyzes MHET into TPA and EG, which are subsequently metabolized through central metabolic pathways (Yoshida et al., 2016). Polyethylene presents additional challenges due to its non-polar, high-molecular-weight structure. Its degradation is initiated by oxidation, mediated by enzymes such as laccases or peroxidases, which introduce functional groups like hydroxyl or carboxyl groups. These oxidized intermediates are then cleaved by hydrolases into smaller oligomers and monomers (Restrepo-Flórez et al., 2014).

In recent years, biodegradation has gained traction as a waste management strategy, driven by increased understanding of the requirements for degrading various polymers. Many plastics contain additives that enhance resistance to microbial attack, necessitating highly specific enzymes to penetrate the polymer matrix. Given that widespread plastic pollution has only occurred over the past seven decades, there has been limited evolutionary time for the development of such enzymes. While the fundamental principle involves breaking down polymers into mono- or dimers for further metabolism, factors such as hydrophobicity, strong chemical bonds, high molecular weight, and the presence of antimicrobial additives can significantly prolong the biodegradation process (Danso et al., 2019).

In this study, the main objective was to isolate and characterize a bacterial strain from Icelandic soil capable of degrading PET. The research focused on evaluating the growth of the strain in minimal media with plastics as the sole carbon source. Furthermore, proteomic analyses were performed to elucidate the cellular mechanisms that enable this strain to survive and metabolize PET under these conditions. Ultimately, the study aimed to contribute to the broader understanding of microbial plastic degradation, especially in scenarios where enzymatic degradation is not clearly observed.

## 2 Materials and methods

**FIGURE 1:**
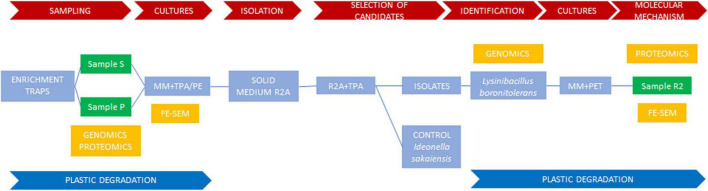
Summary of the overall experimental strategy. Schematic representation of the experimental design followed to isolate and characterize a bacterial strain from Icelandic soil capable of degrading PET.

A summary of the overall experimental strategy is represented in Figure 1.

### 2.1 Study site and sample collection

A site was selected in North Iceland for targeted sampling aimed at isolating bacteria with potential plastic-degrading capabilities. The location, near the University of Akureyri (coordinates: 65.682217, −18.121422), was used to establish experimental “enrichment traps,” where PE plastic fragments were embedded into the soil. These traps were designed to promote microbial adaptation to plastic substrates over time. Sampling was carried out after 5 months, in the winter season, as organic decomposition is known to continue beneath the snow cover in subarctic climates.

Two distinct sample types were collected from the enrichment traps: a soil sample, referred to as sample S, and a plastic-associated sample, designated as sample P. For each sample type, three biological replicates were analyzed at each sampling point.

### 2.2 Bacterial isolation

To initiate the enrichment process, 15 g of soil were suspended in 85 mL of sterile mineral medium (MM), supplemented with either 100 mg of TPA or PE granules. The PE used was analogous to the material previously buried in enrichment traps, but prepared under sterile laboratory conditions. Cultures were incubated at room temperature for a period of 7 to 14 days, with microbial growth monitored every three days by plating on R2A agar.

Mineral media promotes plastic degradation by limiting easily metabolizable carbon sources, pushing bacteria to use PET as the primary carbon source. It contains sodium acetate and ammonium sulfate for balanced growth, along with essential trace minerals like Fe^2+^, Mn^2+^, and Mo^6+^ that support enzymatic activity. B vitamins enhance bacterial metabolism and PET breakdown. Phosphate buffering maintains pH stability, while magnesium and sulfate ions aid enzymatic function. Designed to mimic nutrient-limited environments, mineral media offers more realistic biodegradation conditions than traditional mineral medium (MM), which lacks key nutrients and requires external carbon supplementation, potentially affecting experimental consistency and bacterial adaptation (Schneier et al., 2024).

All enrichment and isolation procedures were performed in triplicate to ensure reproducibility. Control cultures, consisting of the same mineral medium and inoculum but lacking TPA or PE, were included to differentiate background microbial activity from plastic-induced responses. A noticeable decline in microbial growth within the enrichment medium was used as an indicator to proceed with further isolation and characterization.

### 2.3 16 S rRNA sequencing of the isolated samples

16S rRNA sequencing identified *Lysinibacillus* sp. RBD_002 as the dominant bacterial species in the Icelandic soil sample, with 80,825 reads. Minor ASVs corresponding to Bacillus licheniformis and an unidentified Bacillaceae were present at much lower abundances. Taxonomic profiling confirmed the identity of *Lysinibacillus* sp. RBD_002, supporting its selection for further biodegradation studies involving PET and TPA.

### 2.4 Screening of bacterial isolates for terephthalic acid (TPA) utilization

To evaluate the capacity of isolated bacterial strains to metabolize TPA, a two-step screening was performed on solid R2A medium supplemented with TPA (1,000 mg/L, dissolved in DMSO). Cultures were applied in triplicate to ensure reproducibility and statistical reliability.

Following enrichment, individual strains were isolated through selective plating and colony purification. Enrichment cultures (1 mL) were plated on R2A agar overlaid with TPA, providing selective pressure for microorganisms capable of tolerating or utilizing PET-derived intermediates. Colonies that appeared on TPA plates (TPA1) were streaked onto fresh TPA plates (TPA2) to obtain purified isolates. Strains demonstrating consistent growth on both TPA1 and TPA2 were considered candidate PET-associated bacteria and were transferred to liquid mineral medium containing PET as the sole carbon source. 16S rRNA gene sequencing and ASV analysis confirmed that *Lysinibacillus* sp. RBD_002 was the dominant isolate recovered after selective enrichment and purification.

### 2.5 Evaluation of PET degradation via OD600 growth measurements

Bacterial growth was monitored using optical density at 600 nm (OD600) in liquid cultures containing PET plastic as the sole potential carbon source. The aim was to assess whether the isolated *Lysinibacillus* strain could utilize PET for growth, with *I. sakaiensis* serving as a positive control due to its known PET-degrading capabilities (Yoshida et al., 2016). All cultures were standardized to an initial OD600 of 1.00 and prepared in triplicate to ensure reproducibility and statistical robustness.

Two media types were employed: 10% R2A, a low-nutrient medium suitable for oligotrophic bacteria, and MM, a defined medium commonly used in plastic degradation studies. Each culture contained pre-sterilized PET pieces as the sole or primary carbon source. OD600 readings were taken weekly over six weeks using a CLARIOstar microplate reader (BMG LABTECH) to track changes in cell density, which may indicate bacterial adaptation and PET utilization.

Growth patterns were compared across media types and against the *Ideonella* control to evaluate metabolic flexibility and stress adaptation associated with plastic degradation. No additional carbon sources were added, ensuring that any observed growth was attributable to PET or trace nutrients in the media. This approach aligns with methodologies used in prior studies which employed OD600 measurements to assess microbial growth in the presence of synthetic polymers over extended incubation periods (Urbanek et al., 2018).

### 2.6 Field emission scanning electron microscopy (FE-SEM)

PET plastic 1.5 cm × 1.5 cm was placed into the bacterial cultures for four weeks in 30 °C. Two plastic pieces were cut out of a plastic bottle, sterilized with ethanol, dried for 24 h, and sterilized again under the UV light for 60 min. One additional plastic piece was prepared as well and was later used as a negative control. Two plastic pieces mentioned above were placed into the culture flasks with the bacterial samples. One pieces with the bacteria from the soil and one piece with the bacteria from the plastic waste dump. Negative control plastic piece was placed to a closed petri dish and was stored there for four weeks until the electron microscope examination. After four weeks the plastic pieces were looked at under the electron microscope in different magnifications. Microorganisms on plastic samples were observed by FE-SEM, using a ThermoScientific (Boston, MA, USA) APREO C-LV microscope, with an AZTEC ORFORD energy dispersive X-ray (EDX) microanalysis system. The samples were coated with 4 nm of chromium using a sputtering Leica EM ACE 600. The images were obtained at 10 kV (Hidalgo-Arias et al., 2023).

These analyses were carried out in two distinct phases. In the first phase, experiments were conducted using original environmental soil samples that had been incubated with sterilized PET plastic strips at 30 °C for four weeks. The aim was to screen for early signs of microbial colonization or degradation activity associated with the native microbial community. In the second phase, following the isolation of a promising bacterial candidate, *Lysinibacillus*, the experiment was repeated using this pure culture. PET fragments were incubated with the bacterial strain in liquid mineral medium under similar conditions (30 °C for six weeks). This approach enabled a comparative assessment between community-level interactions and the activity of the isolated strain. A negative control PET sample, not exposed to any microbial culture, was stored in a sterile Petri dish for the same duration and analyzed in parallel to distinguish biological effects from abiotic changes on the polymer surface.

All PET samples were examined under various magnifications to evaluate surface topography, biofilm formation, and potential indicators of degradation, such as pitting, cracking, or erosion.

### 2.7 DNA extraction and metabarcoding data processing

Genomic DNA was extracted from the environmental samples using the FastDNA™ SPIN Kit for Soil (MP Biomedicals), following the manufacturer’s protocol with minor adjustments. Mechanical disruption of microbial cells was performed using the FastPrep^®^ instrument. DNA concentration was quantified using the Qubit™ fluorometric system (Garcia-Lopez et al., 2022).

The sequencing procedure was performed on Illumina MiSeq 2 × 250–2 × 300, to obtain approximately 100,000 reads per sample. The amplification and sequencing of the V3-V4 regions of the 16S rRNA gene (forward sequence CCTACGGGNGGCWGCAG; reverse sequence GACTACHVGGGTATCTAATC) were performed to identify bacteria (García-Lopez et al., 2021).

Data analysis was performed using Qiime2 (v2024.10) (Bolyen et al., 2019) to process the reads obtained from the sequencing and acquire taxonomic results. The primers from 16S rRNA amplicons were removed with the CUTADAPT tool (via q2-cutadapt) (Martin, 2011). Then, reads were processed with DADA2 (via q2-dada2) (Callahan et al., 2016) to obtain the representative sequences and the amplicon sequence variants (ASV) table using the following parameters: –p-trunc-len-f 245 –p-trunc-len-r 196 for 16S rRNA amplicons. Taxonomic annotations were performed using the classify-sklearn method from the feature-classifier Qiime2 plugin. Naïve-Bayes trained classifiers were used in this step. SILVA reference database was used to train the classifiers (release 138-99-nr).

### 2.8 *In silico* screening for polyester hydrolases

At present, no experimentally verified PETase has been reported in *Lysinibacillus*. Therefore, a broad motif-guided approach was adopted to capture distant homologues of cutinases, lipases, and PhaZ enzymes potentially capable of polyester hydrolysis. The search focused on members of the α/β-hydrolase superfamily, as this fold is widely conserved among microbial cutinases, lipases, and polyesterases. These enzymes typically exhibit a Ser-His-Asp/Glu catalytic triad and the conserved GXSXG nucleophilic motif, which together with an oxyanion-stabilizing loop constitute the principal sequence signatures used for polyesterase detection. The relevance of these structural motifs for identifying cutinase-like and polyester-hydrolyzing enzymes has been well established in comparative and structural studies of cutinases and related α/β-hydrolases (Nikolaivits et al., 2018). Because polyester hydrolysis is not restricted to PETase-like enzymes, additional searches were carried out for polyhydroxyalkanoate depolymerases (PhaZ) and lipases/esterases known to act on aliphatic polyesters such as PLA and PCL, as these enzyme classes share the same α/β-hydrolase catalytic architecture and the GXSXG motif (Nikolaivits et al., 2018).

Polyester hydrolase genes were searched in the genome of *Lysinibacillus* sp. based on the complete genome available at NCBI.^1^ The catalytic motif known as the “lipase box,” represented by the sequence GXSXG (where serine acts as the nucleophile), was targeted for identification. Verification included checking for histidine, aspartate, or glutamate residues in proximity, as well as the oxyanion pocket motif (HGG) near the serine residue. Prioritization was based on the likelihood of secretion, indicated by a predicted signal peptide at the N-terminus, which is characteristic of extracellular cutinases and lipases, along with considerations of compatible size.

#### 2.8.1 Genome-mining methods

Predicted proteomes or genomes were first prepared by generating annotated protein sets from input FASTA files using standard prokaryotic annotation pipelines. When starting from nucleotide sequences, annotation was performed with widely used genome annotation frameworks, which provide high-quality predicted proteins and associated HMM models.

Subsequently, a similarity-based search strategy was applied using both BLASTp and HMMER. Sets of well-characterized polyester-degrading enzymes were compiled as reference seeds, including PETases and cutinase-like hydrolases such as cutinases and engineered polyesterases, whose structural and catalytic features have been described extensively, including the conserved α/β-hydrolase fold, the GXSXG motif, and the Ser-His-Asp/Glu catalytic triad. These structural markers are highlighted in recent cutinase and polyesterase reviews (Kumar et al., 2024). Multiple sequence alignments of seed sequences were used to build HMM profiles for hmmsearch, and domain models corresponding to cutinase/lipase families within the α/β-hydrolase fold were employed to detect distant homologues. BLASTp searches were conducted using conservative thresholds (identity ≥ 25%–30%, coverage ≥ 60%, e-value ≤ 1e-10) to produce an initial shortlist of candidates (Nikolaivits et al., 2018).

Candidate hits were then filtered for the presence of catalytic and structural motifs characteristic of polyester hydrolases, including the GXSXG nucleophilic motif and the oxyanion-stabilizing loop typical of α/β-hydrolases. Additional features associated with extracellular polyesterases, such as predicted secretion signals, disulfide-bond-forming cysteines, and compatible α/β-hydrolase domains, were assessed based on criteria outlined in recent structural and biochemical analyses of cutinases and polyesterases (Nikolaivits et al., 2018).

Finally, candidate prioritization was carried out by integrating multiple lines of evidence, including the presence of α/β-hydrolase domains, correct catalytic architecture, and similarity to high-performance polyesterases reported in recent molecular and structural studies. These analyses also considered the broader phylogenomic landscape of bacterial cutinase-like enzymes that has been revealed by recent large-scale surveys of PET-active hydrolases (Kumar et al., 2024).

All analyses were performed with standard command line HMMER and BLAST+ tools. Scripts used for the searches (bash wrappers for hmmsearch and blastp) and the downstream filtering (Python notebook for motif detection and annotation merging) can be supplied to enable full reproducibility of the workflow.

### 2.9 Protein extraction

Both S samples and P samples were analyzed using proteomics techniques. In addition, the proteome of *Lysinibacillus* cultivated in minimal media with plastics as the sole carbon source, named sample R, was analyzed.

Samples were individually transferred to sterile 50 mL Corning tubes and centrifuged at 8,000 × *g* for 10 min. The supernatant was decanted and replaced with cold phosphate-buffered saline (PBS, 4 °C), followed by a second centrifugation under the same conditions. The pellet was resuspended in 3 mL of TIIP buffer (protease inhibitor isotonic buffer), ensuring complete homogenization without clumps. For mechanical lysis, the resuspended cells were aliquoted into cryotubes containing glass beads. Lysis was performed using the FastPrep MP system, following the Evaprot protocol. The lysates were centrifuged at 13,000 rpm for 3 min using an Eppendorf centrifuge. The supernatant, containing soluble proteins, was carefully collected for downstream analysis. All steps were carried out at 4 °C. Protein concentration was quantified using the Qubit™ Protein Assay Kit (Ruiz-Blas et al., 2023).

### 2.10 Protein identification

Proteins were analyzed using 2-dimensional electrophoresis (2-DE) by combining horizontal slab gel isoelectric focusing (IEF) with SDS-PAGE. Carrier ampholyte urea IEF was performed using pH 4–7 strips (11 cm). The spots resolved by 2-DE from the gels were stained with Coomassie Blue for peptide mass fingerprinting or MS/MS analysis and protein identification. Spectral data were analyzed to search them using the Mascot search engine (Matrix Science, London, UK). Search parameters were: Database: NCBI; Taxonomy: Eubacteria; Enzyme: Trypsin; Fixed modifications: Carbamidomethyl (C); Variable modifications: Oxidation (M); Mass values: monoisotopic; Protein Mass: Unrestricted; Peptide Mass Tolerance: ± 80 ppm; Fragment Mass Tolerance: ± 0.3 Da; Max Missed Cleavages: 1; Instrument type: MALDI-TOF-TOF (Martinez-Alonso et al., 2019). The mass spectrometry proteomics data have been deposited in the ProteomeXchange Consortium^2^ via the PRIDE partner repository (Perez-Riverol et al., 2019) with the dataset identifier PXD064056. Protein functions were searched for in UniProt.^3^

## 3 Results and discussion

### 3.1 Bacterial community composition of the soil sample

**FIGURE 2:**
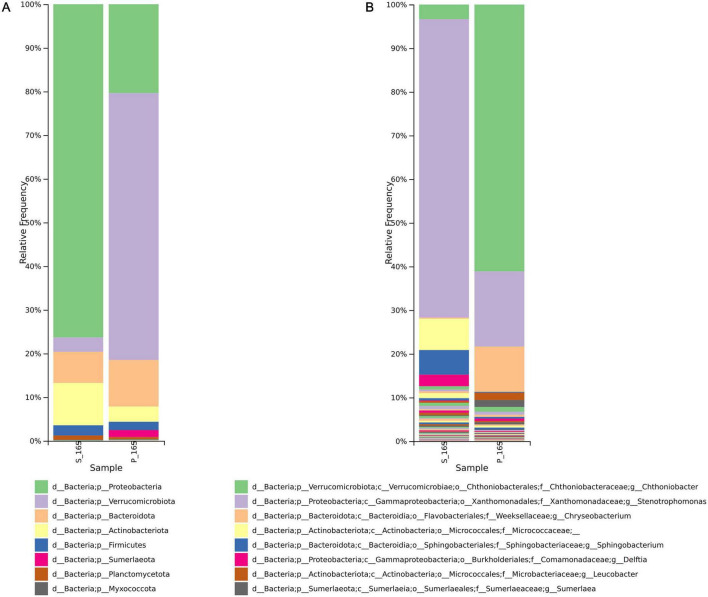
Barplots representing the microbial community distribution in the Icelandic soil samples. Relative abundances of bacteria at the phylum **(A)** and genus **(B)** levels based on 16S rRNA gene sequencing data. The detailed taxonomy is summarized in Supplementary Table 2.

Amplicon sequencing of the 16S rRNA gene from the environmental soil sample revealed a taxonomically rich and diverse microbial community (Figure 2 and Supplementary Table 2).

The dominant bacterial phyla in the soil sample S were Proteobacteria (76%), Actinobacteriota (10%), and Bacteroidota (7%), which are commonly associated with soil ecosystems and known for their functional versatility (Figure 2A). The dominant bacterial phyla in sample P were Verrucomicrobiota (61%), Proteobacteria (20%), and Bacteroidota (11%).

At the genus level, the bacterial communities identified in the soil and plastic samples exhibited marked differences in composition (Figure 2B). In the soil sample (S), the genus *Stenotrophomonas* was predominant, whereas the sample P was mainly characterized by the presence of *Chitinobacter*. Notably, *Lysinibacillus*, a genus within the Firmicutes phylum, was also detected, albeit in lower relative abundance. Its presence is particularly relevant to this study, as it was later isolated and investigated for its potential role in PET plastic degradation.

The composition of the microbial community indicates a metabolically diverse consortium, with several genera previously implicated in biodegradation processes, stress response, and environmental resilience.

### 3.2 Proteomic analysis of the environmental soil sample

**FIGURE 3:**
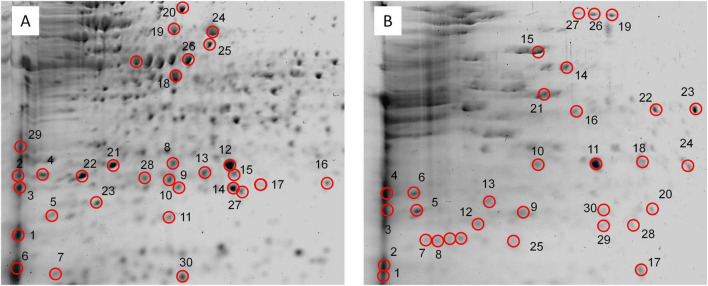
Proteins from soil samples resolved by 2-DE. Numbered spots marked with circles corresponded to proteins identified by MALDI-TOF and described in Supplementary Table 3. **(A)** soil sample, referred to as sample “S.” **(B)** plastic-associated sample, designated as sample “P.” The figure is representative of three 2-DE experiments.

Proteomic analysis of the whole soil sample revealed a complex protein profile, indicating active microbial metabolism (Supplementary Table 3 and Figure 3). Several proteins associated with stress response and potential plastic interaction were detected, including peroxiredoxin, thiol peroxidase, Fe-Mn superoxide dismutase, ATP-dependent Clp protease, and universal stress proteins. These enzymes are commonly linked to oxidative stress regulation and survival under toxic or nutrient-limited conditions.

A VOC (Vicinal Oxygen Chelate) protein from *Lysinibacillus* was identified in soil cultures. Some VOC proteins, especially estradiol dioxygenases, are involved in the degradation of aromatic compounds, which are structurally similar to certain plastics such as PET and PS. The fact that this bacterium contains VOC proteins suggests that it could be involved in the biodegradation of aromatic plastics (Jamal and Ahmad, 2022).

A *Pseudomonas* amidohydrolase was also identified. This enzyme catalyzes the hydrolysis of amide, ester, phosphate, and other bonds in organic compounds. Some amidohydrolases can break ester bonds, which are present in plastics such as PET, PLA, and PCL (Sugrue et al., 2015).

*Shinella* phage tail protein was identified. Some of these proteins, especially tail spike proteins, have depolymerase enzymatic activity, which allows them to degrade polysaccharides on the bacterial surface (Knecht et al., 2020). *Shinella* has demonstrated the ability to degrade 1,4-dioxane, a persistent organic pollutant. This suggests potential for biodegradation, but not specifically of plastics such as PET or PE (Tian et al., 2023).

The NAD(P)H: quinone oxidoreductase enzyme in *Enterobacter* does not degrade plastics directly. However, *Enterobacter* has demonstrated the ability to degrade PE and PU, especially through alkane monooxygenase. NAD(P)H: quinone oxidoreductase could participate in associated redox pathways, but it is not the main enzyme (Mohamed and Narayanan, 2024).

Superoxide dismutase (SOD) was identified from *Pseudomonas*, *Lysinibacillus*, and *Buttiauxella*. SOD does not degrade plastic directly, but it can facilitate the process by reducing oxidative stress (Esmaeili et al., 2013).

Additionally, a few hypothetical proteins were detected, including HMPREF0490_00663 from Lachnospiraceae bacterium 6_1_37FAA, suggesting unknown but potentially relevant biological functions in plastic-rich environments.

### 3.3 Isolation of PET-degrading bacterial candidates from environmental soil sample

The isolation of potential plastic-degrading bacteria was achieved using solid R2A media with a top layer containing TPA dissolved in DMSO. This selective culturing approach favored microorganisms capable of utilizing TPA as a carbon source, allowing for the targeted recovery of candidates with potential plastic biodegradation capabilities. Among the isolates, a bacterial strain *Lysinibacillus* sp. RBD_002 was identified. The selective setup was designed to promote the growth of microorganisms capable of utilizing TPA as a carbon source. Colonies that successfully grew under these conditions were considered strong candidates for potential PET biodegradation and were selected for further testing.

Colonies were cultivated in liquid mineral media supplemented with PET plastic strips. Visible possible microbial accumulation was observed at the interface between the PET and the bottom of the flask. These observations were consistent across replicates and coincided with OD600 measurements indicating microbial growth.

### 3.4 Bacterial growth dynamics in PET-containing medium

**FIGURE 4:**
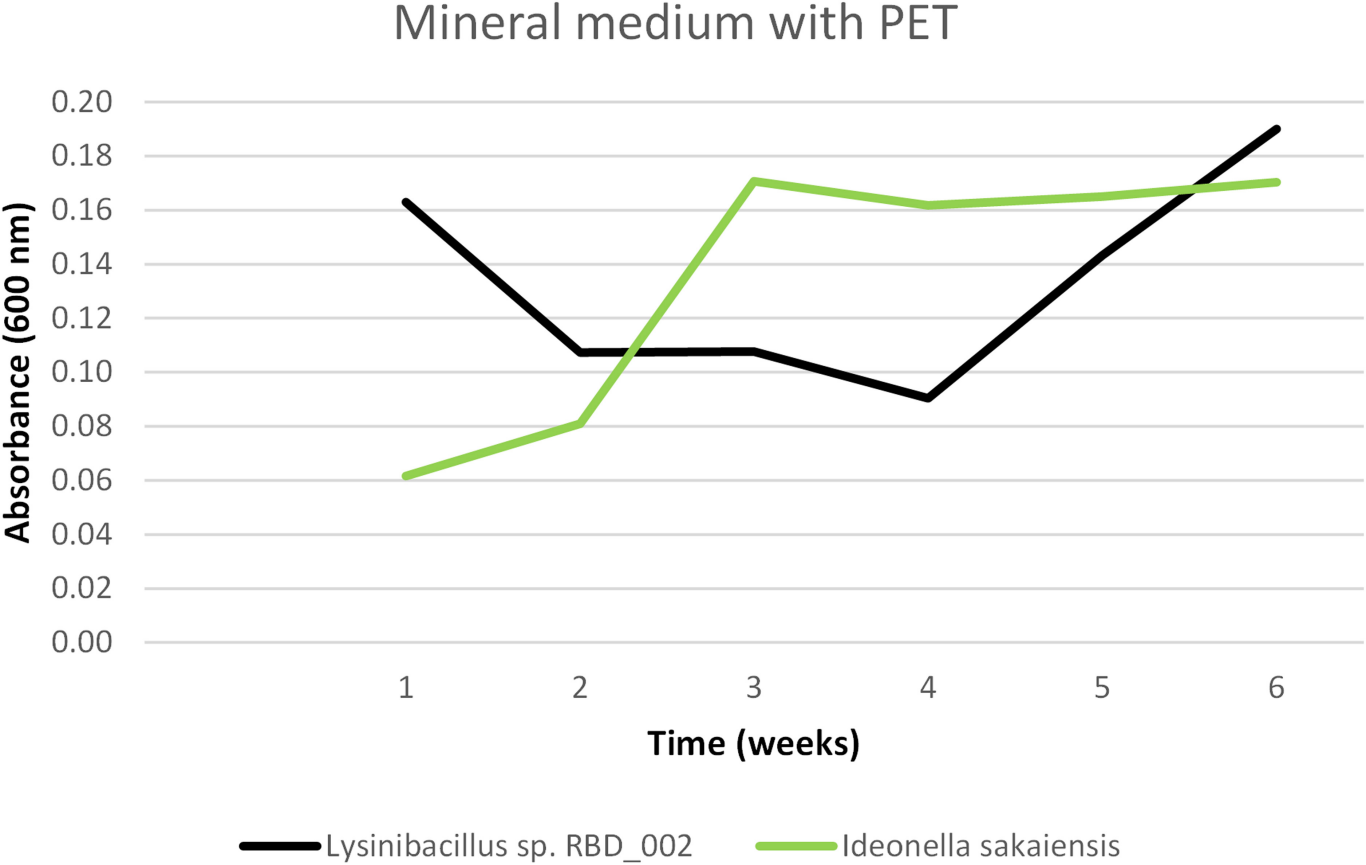
Comparison of OD600 values for *Lysinibacillus* and *Ideonella* in mineral medium with PET over six weeks. *Lysinibacillus* exhibited a shorter lag phase and faster initial growth, while *Ideonella* showed steady, gradual proliferation. These trends suggest differential adaptation strategies to PET-derived carbon and highlight the potential of *Lysinibacillus* for early-stage plastic degradation.

Optical density measurements at 600 nm (OD600) were employed to monitor bacterial growth dynamics in liquid media containing PET plastic as the sole carbon source (Figure 4). The results revealed a consistent increase in OD600 values over time, indicating active proliferation of the bacterial strains. Notably, the isolated strain *Lysinibacillus* sp. RBD_002 demonstrated a shorter lag phase and more rapid adaptation to the PET-containing environment compared to the positive control strain, *I. sakaiensis*. While *I. sakaiensis* exhibited steady growth, *Lysinibacillus* sp. RBD_002 adapted more quickly, suggesting the presence of mechanisms that facilitate early-stage utilization of PET-derived carbon or confer enhanced tolerance to stressors associated with plastic degradation. These findings highlight the potential of *Lysinibacillus* sp. RBD_002 as a candidate for biotechnological applications in plastic biodegradation.

Furthermore, an independent samples *t*-test was performed to assess whether the differences in mean OD600 values between *Lysinibacillus* sp. RBD_002 and *I. sakaiensis* were statistically significant at each timepoint. A *p*-value below 0.05 was considered statistically significant. OD600 readings were collected weekly over a six-week period, with both strains grown in mineral medium containing PET plastic as the sole carbon source. The *t*-test revealed significant differences in growth at Week 1 (*p* = 0.0002), Week 2 (*p* = 0.0214), Week 4 (*p* = 0.0011), and Week 6 (*p* = 0.0044). No significant differences were observed at Week 3 (*p* = 0.1630) and Week 5 (*p* = 0.3321). These results suggest that *Lysinibacillus* initially exhibited higher OD600 values, followed by a plateau during mid-incubation. By Week 4, *I. sakaiensis* surpassed *Lysinibacillus* in growth, with both strains showing similar OD600 values in Week 5. At the final timepoint, *Lysinibacillus* demonstrated a renewed growth increase that was statistically significant. Overall, the data reveal dynamic, time-dependent growth patterns, highlighting differential responses to PET as a carbon source.

### 3.5 Plastic degradation by *Ideonella* and *Lysinibacillus*

**FIGURE 5:**
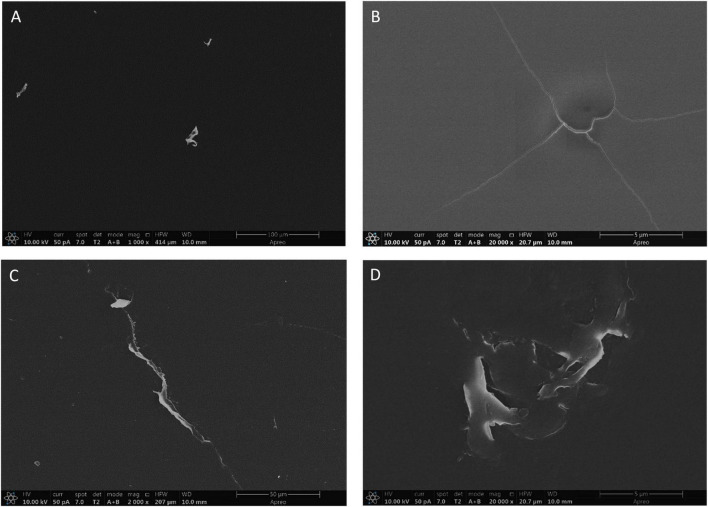
FE-SEM images of PET plastic strips. **(A)** Negative control (unexposed to bacterial culture), captured at 1000× magnification. **(B)** Negative control at 20000×. **(C)** PET plastic exposed to environmental soil bacteria, 2000×. **(D)** PET plastic exposed to environmental soil bacteria, 20000×.

FE-SEM imaging provided high-resolution visualization of microbial colonization on PET plastic surfaces exposed to environmental soil samples (Figure 5). Distinct structural modifications were observed in several regions, including surface pitting, erosion patterns, and irregular depressions, features that suggest active microbial degradation. In contrast, control samples retained smooth, unaltered surfaces, indicating the absence of such biological activity of PET plastic exposed to environmental soil bacteria (Figure 5).

**FIGURE 6:**
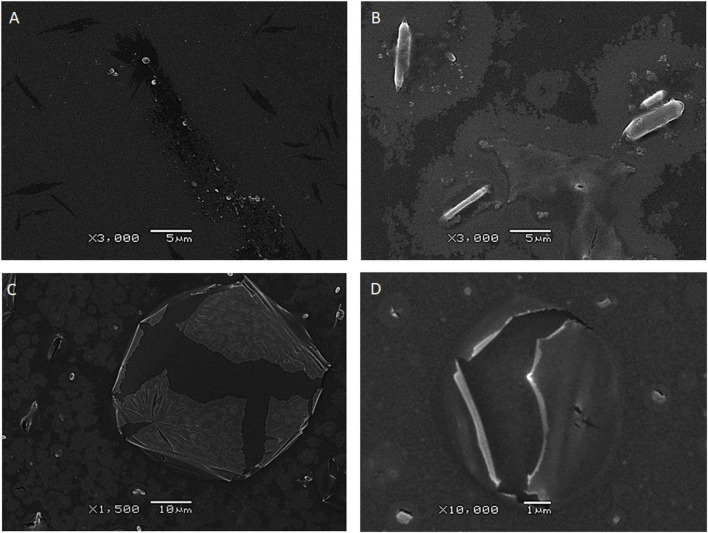
FE-SEM imaging of PET samples incubated with *Lysinibacillus* and *Ideonella*. **(A)** Control PET strips showed smooth surfaces with no signs of microbial colonization. **(B)** PET exposed to *Lysinibacillus* revealed rod-shaped bacterial cells. **(C)** PET exposed to *Lysinibacillus* showed surface damage, including fissures and peeling layers, indicative of enzymatic degradation. **(D)** PET incubated with *Ideonella* displayed surface cracking and delamination, consistent with microbial interaction and polymer breakdown.

PET samples incubated with the isolated bacterial strains also provided direct visual evidence of microbial activity (Figure 6). Surface alterations such as erosion patterns, biofilm formation, pits, and cavities were observed, indicating colonization and degradation of the polymer. In contrast, control PET strips remained largely intact, supporting the role of bacterial action in structural changes. In the negative control images (Figure 6A), the PET surface appeared smooth and undisturbed, with minor particulates likely due to preparation artifacts under sterile conditions. PET samples exposed to *Lysinibacillus* showed rod-shaped structures consistent with bacterial cells attached to the surface (Figure 6B), suggesting active colonization. Pronounced morphological changes, including central fissures and peeling layers, were evident, likely resulting from enzymatic degradation (Figure 6C). Similarly, PET exposed to *I. sakaiensis* exhibited distinct surface cracking and delamination, characteristic of enzymatic breakdown, confirming active interaction between the strain and the polymer substrate (Figure 6D).

### 3.6 Combined proteomic and *in silico* insights into plastic degradation mechanisms in *Lysinibacillus*

Proteomic analysis using MALDI-TOF mass spectrometry was conducted to identify proteins expressed by *Lysinibacillus* when cultured with PET as the sole carbon source (Supplementary Table 4 and Supplementary Figure 1). Mascot-based database searches did not reveal canonical PET-degrading enzymes such as PETase or MHETase, which have been previously identified in *I. sakaiensis* (Yoshida et al., 2016; Kim et al., 2020). However, the dataset predominantly contained proteins associated with stress responses, central metabolism, and general hydrolase activity.

Among the hydrolases detected, maleamate amidohydrolase, an enzyme acting on C-N bonds in linear amides, was identified. While not directly linked to plastic degradation, its activity on amide bonds suggests potential relevance for breaking down polymers such as nylon or aromatic polyurethanes (Pérez-García et al., 2025). However, its presence cannot be taken as evidence of PET depolymerization, as this enzyme type is not known to cleave ester bonds or degrade PET-like aromatic polyesters. Peptide methionine sulfoxide reductase, a protein repair enzyme associated with oxidative stress mitigation, was also detected but does not participate in polyester hydrolysis (Aussel and Ezraty, 2021). Similarly, phosphoadenosine 5′-phosphosulfate reductase and tautomerase YrdN may modulate cellular physiology or interact with stress-related intermediates, yet neither enzyme has any documented role in PET degradation or in the hydrolysis of synthetic polyesters (Kamp, 2013; Fu et al., 2018). Collectively, these hydrolase annotations reflect general metabolic and stress-response functions rather than direct involvement in PET depolymerization. In line with validated PET hydrolases-typically serine hydrolases characterized by the GxSxG motif, a Ser-His-Asp catalytic triad, and a compatible active-site cleft, none of the detected proteins displayed the canonical sequence signatures required for polyesterase activity.

A potential involvement of laccase (EC 1.10.3.2) was considered because laccases have been reported to oxidize a variety of aromatic substrates and certain synthetic polymers (Pérez-García et al., 2025). However, in this study laccase was not directly identified in the MALDI-TOF proteome, as the characteristic peptide masses for this enzyme fall outside the detection window of the analytical range typically used (500–4,000 Da). Therefore, no laccase peptides, annotations, or activity-derived signatures could be confirmed experimentally. Although some studies have suggested that laccases may contribute to surface oxidation of aromatic plastics, direct and efficient PET depolymerization by laccases alone has not been conclusively demonstrated, and such activity was not validated under our experimental conditions. Consequently, any potential role of laccase in PET surface modification must be regarded as speculative, and no functional contribution can be inferred from the present data.

In addition to these enzymes, several proteins associated with oxidative stress responses were identified, including peroxiredoxins, superoxide dismutases, thiol peroxidases, and ATP-dependent Clp protease subunits. These proteins help mitigate oxidative damage and support cellular survival under chemically challenging conditions (Supplementary Table 3 and Figure 3). Furthermore, a number of hypothetical and uncharacterized proteins were detected. While their functions remain unknown, their expression under PET exposure suggests involvement in the cellular adaptation to plastic-derived stressors.

From the metaproteome, several molecular mechanisms underlying plastic degradation in *Lysinibacillus* can be inferred. The bacterium appears to undergo broad metabolic reprogramming, with upregulation of central metabolic pathways such as the TCA cycle, which are essential for processing intermediates derived from plastic substrates (Pérez-García et al., 2025).

Among the proteins involved in the TCA cycle, fumarate reductase subunit C plays a role in the conversion of fumarate to succinate, a reaction particularly relevant under anaerobic conditions (Weingarten et al., 2009). Succinate-CoA ligase subunit β is a key enzyme in the TCA cycle, catalyzing the transformation of succinyl-uccinylnsformation of succinycle, rly relevant under anaerobic conditions ditions icula (Schneider et al., 1998). Although ATP synthase gamma chain and ATP synthase subunit b are not directly part of the TCA cycle, they are essential for ATP synthesis driven by the proton gradient generated by the electron transport chain (Guo et al., 2019). Finally, ferredoxin-3 contributes to electron transfer in various metabolic pathways, including those associated with the TCA cycle, by facilitating redox reactions through its iron-sulfur clusters (Terranova, 2024).

Concurrently, the expression of membrane transport systems is modulated to facilitate the uptake of plastic-derived molecules. The chemical stress imposed by PET exposure also activates oxidative stress mitigation systems, allowing the cell to manage toxic byproducts generated during plastic metabolism (Herrera et al., 2023; Heris, 2024). These adaptations reflect a shift in cellular resource allocation, redirecting energy from growth toward survival and stress tolerance under nutrient-limited and contaminant-rich conditions (Herrera et al., 2023).

#### 3.6.1 *In silico* screening of candidate polyester hydrolases

In parallel with the proteomic study, an *in silico* screening for catalytic motifs was conducted to explore the genetic potential for polyester hydrolysis. A total of 4,497 proteins (≈1.29 million amino acids) were analyzed for the presence of the GXSXG nucleophilic motif. Among these, 398 proteins contained the motif (Supplementary Table 5). After applying filters for proximal catalytic signals (HGG oxyanion pocket), nearby histidine/aspartate/glutamate residues that may form a catalytic triad, and secretion likelihood, 17 strong candidates remained. Supplementary Table 6 highlights the four highest-priority candidates, each predicted to possess both a secretion signal and the GXSXG motif.

However, none of these four proteins matched the proteins detected in the MALDI-TOF proteome. Moreover, none of the 17 candidates has been reported in the literature as a functional polyester hydrolase or plastic-degrading enzyme. Therefore, no canonical plastic-degrading enzymes were detected under the tested conditions; this absence reflects the expression state under PET exposure rather than the genetic absence of such enzymes.

Overall, the combined proteomic and *in silico* results indicate that PET transformation in *Lysinibacillus* is associated with global metabolic adaptation and stress-response remodeling rather than with the expression of specialized PET-degrading enzymes. Unlike microorganisms that synthesize dedicated PETase- or MHETase-like hydrolases, *Lysinibacillus* appears to reorganize its core metabolism, stress-mitigation systems, and possibly oxidative surface-modifying enzymes when PET is provided as the sole carbon source. Such adaptation may indirectly support limited PET transformation as a consequence of metabolic flexibility and stress-driven remodeling rather than via specific ester-bond-cleaving polyesterases.

## 4 Conclusion

In *Lysinibacillus*, plastic degradation does not occur through the production of dedicated plastic-degrading enzymes. Instead, when plastic is the sole carbon source available, the bacterium undergoes a global reorganization of its cellular machinery. This adaptation involves the modulation of metabolic pathways to redirect energy and resources toward the assimilation and breakdown of plastic-derived compounds.

Since *Lysinibacillus* exhibited a shorter lag phase and faster initial growth, while *I. sakaiensis* showed a steady and gradual proliferation, these trends suggest distinct adaptation strategies to PET-derived carbon sources. Notably, the rapid response of *Lysinibacillus* highlights its potential for early-stage plastic degradation. These complementary characteristics suggest that a microbial consortium combining both species could be advantageous, leveraging their distinct plastic-degrading capabilities throughout the entire degradation process.

## Data Availability

Sequences obtained by 16S rRNA sequencing were deposited in the NCBI Short Read Archive (SRA), BioProject PRJNA1097487. Biosamples: SAMN40910214, SAMN40910215, SAMN40910232, and SAMN40910233. The mass spectrometry proteomics data have been deposited to the ProteomeXchange Consortium via the PRIDE partner repository with the dataset identifiers PXD068661 (samples S and P) and PXD068590 (sample R2) (Perez-Riverol et al., 2019).

## Footnotes

1
https://www.ncbi.nlm.nih.gov/datasets/genome/GCF_002200915.1/

2
http://proteomecentral.proteomexchange.org

3
https://www.uniprot.org/
