# Supplementary material for: Proteomic characterization of Lysinibacillus reveals early-stage PET biodegradation potential
Source: Front Microbiol. 2026 Mar 24;17:1802173. doi: 10.3389/fmicb.2026.1802173 (PMC13053498; doi:10.3389/fmicb.2026.1802173)

Supplementary Material

Proteomic Characterization of *Lysinibacillus* Reveals Early-Stage PET Biodegradation Potential

Radoslaw B. Dudziak[1,2], Víctor Muñoz-Hisado[1,3], Andrea Hidalgo-Arias[1,3], María Martínez-Carrancho[1], Eva Garcia-Lopez[1], Farayde Matta Fakhouri[4], Emma Martinez-Alonso[5], Alberto Alcázar[5], Margrét Auður Sigurbjörnsdóttir[2], Gustavo Graciano Fonseca[2], and Cristina Cid[1]*

1 Centro de Astrobiología (CAB), CSIC-INTA, Carretera de Ajalvir km4, 28850 Torrejón de Ardoz, Madrid, Spain.

2 Faculty of Natural Resource Sciences, School of Business and Science, University of Akureyri, Iceland.

3 Escuela de Doctorado de la Universidad Autónoma de Madrid. Centro de Estudios de Posgrado, Ciudad Universitaria de Cantoblanco, 28049 Madrid, Spain.

4 Department of Materials Science and Engineering, Universitat Politècnica de Catalunya – UPC BarcelonaTech, Terrassa, Spain

5 Departamento de Investigacion, Hospital Ramón y Cajal, Instituto Ramón y Cajal de Investigación Sanitaria, 28034 Madrid, Spain.

*** Correspondence:**
Cristina Cid, Centro de Astrobiología (CSIC-INTA), Ctra. Ajalvir, km 4, 28850 Torrejón de Ardoz, Madrid, Spain
E-mail: cidsc@inta.es; cidsc@cab.inta-csic.es
ORCID: 0000-0001-5128-4558

This file includes:

1. Supplementary Tables. S1-S6.

2. Supplementary Figure. S1.

1 Supplementary Tables

Table S1. Most common commercial plastics and their uses

Polymer	Monomer(s)	Structure	Uses	Reference
PE (LDPE, HDPE)	Ethylene	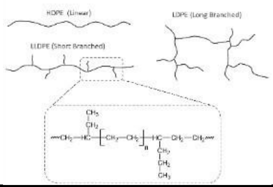	Packaging, thin films	Polymer Database, 2015d
PET	Ethylene glycol, terephthalic acid	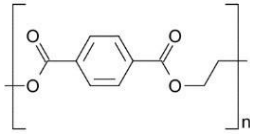	Textile fibres, packaging	Polymer Database, 2015c
PVC	Vinyl chloride	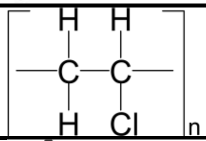	Construction industry	Polymer Database, 2015a
PP	Propylene	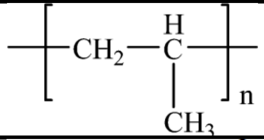	Packaging; the same market as PE	Polymer Database, 2015d
PUR	Organic diisocyanate, diol compound	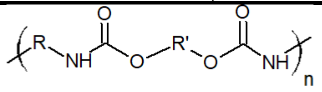 Polyurethane	Foams, sealants, elastomers, adhesives	Polymer Database, 2017
PA	Diacid, diamine	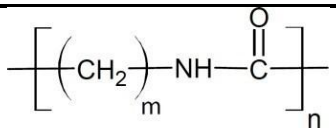	Automotive, engineering industry	Polymer Database, 2015b
PS	Styrene	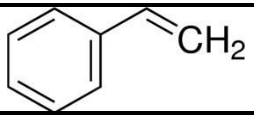	Food industry consumer goods	Polymer Database, 2015e

Table S2. Analysis of bacterial 16S rRNA
Table S2.xlsx

Table S3. Identification of proteins in samples of soil and plastic
Table S3.xlsx

Table S4. Identification of proteins in samples of *Lysinibacillus* cultivated in minimal media with plastics as the sole carbon source
Table S4.xlsx

Table S5. Proteins of *Lysinibacillus* that contain the GXSXG nucleophilic motif
Table S5.xlsx

Table S6. High-priority proteins identified through in silico screening for catalytic motifs. L = length (aa); Motif = "GxSxG"; Pos = position (1-based) of "G"; HGG±100 = presence of HGG at ±100 aa; Secretion = signal peptide.

Protein name	NCBI accession number	L	Motif (Pos)	HGG±100	Secretion	window ±10 aa around the motif
Copper amine oxidase N-terminal domain-containing protein	WP_036080981.1	202	GSSSG (61)	No	Yes	YHNGNGGNNA**GSSSG**GQSYS
TrkH family potassium uptake protein	WP_036075771.1	451	GMSLG (395)	No	Yes	FEITSAFGTC**GMSLG**ITSDL
Beta-propeller domain-containing protein	WP_016992931.1	698	GGSSG (360)	No	Yes	KTTANTKGYL**GGSSG**LYMS
Bifunctional 2',3'-cyclic-nucleotide 2'-phosphodiesterase/3'-nucleotidase	WP_036079417.1	786	GGSYG (697)	No	Yes	FIIITNNYRV**GGSYG**ATFK

2. Supplementary Figures.

Figure S1. Proteins from *Lysinibacillus* samples resolved by 2-DE. Numbered spots marked with circles corresponded to proteins identified by MALDI-TOF and described in Table S4. The figure is representative of three 2-DE experiments.

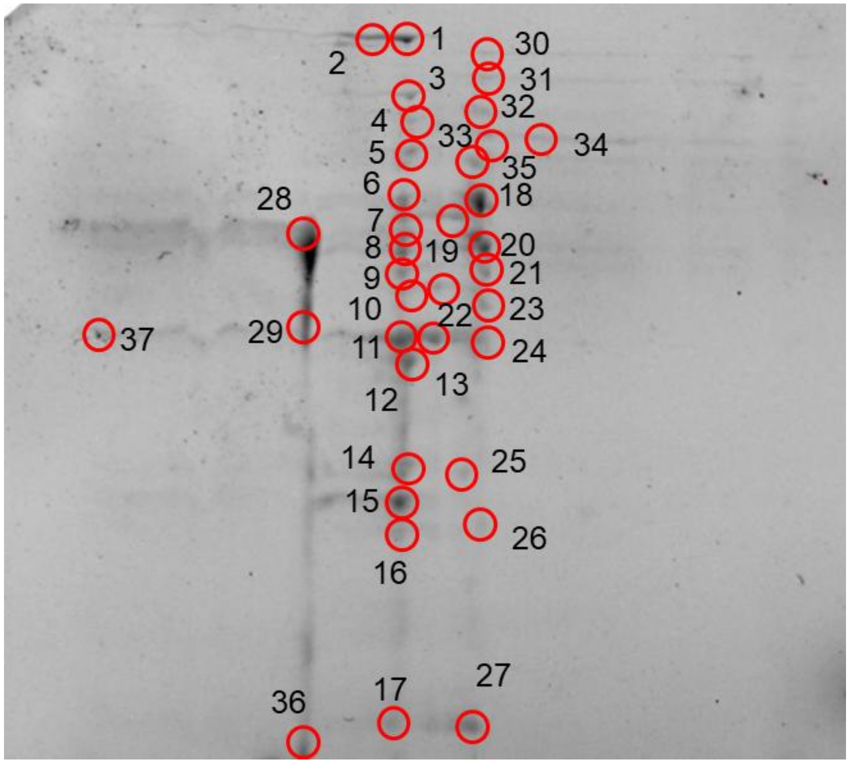

Supplement: Supplementary file 1 [file Data_Sheet_1.PDF]
